# Genetic variants in *TERT* are associated with risk of gastric cancer in a Chinese Han population

**DOI:** 10.18632/oncotarget.13102

**Published:** 2016-11-04

**Authors:** Xianglong Duan, Wei Cao, Lijie Wang, Sida Liu, Zhao Liu, Bolun Zhang, Hua Yang, Tian Feng, Jiayi Zhang, Xiyang Zhang, Yanbin Long, Tianbo Jin

**Affiliations:** ^1^ Department of the Second General Surgery, Shaanxi Provincial People's Hospital, Xi'an 710068, China; ^2^ Department of Oncological Surgery, Shaanxi Provincial People's Hospital, Xi'an 710068, China; ^3^ Department of Respiratory Medicine, Chinese PLA General Hospital, Beijing 100853, China; ^4^ Department of Surgery, Xi'an Chest Hospital, Xi'an TB&Thoracic Tumor Hospital, Xi'an 710100, China; ^5^ Graduate School of Inner Mongolia Medical University, Hohhot 010030, China; ^6^ Xi'an Tiangen Precision Medical Institute, Xi'an, Shaanxi 710075, China; ^7^ School of Life Sciences, Northwest University, Xi'an 710069, China; ^8^ Key Laboratory of Molecular Mechanism and Intervention Research for Plateau Diseases of Tibet Autonomous Region, School of Medicine, Xizang Minzu University, Xianyang, Shaanxi 712082, China

**Keywords:** gastric cancer (GC), telomerase reverse transcriptase (TERT), single nucleotide polymorphisms (SNP)

## Abstract

Telomerase reverse transcriptase (*TERT*) is a gene within the cancer susceptibility region located at Chr5p15.33, which is associated with multiple cancer types. In this study, we validated the association between *TERT* polymorphisms and gastric cancer (GC) risk with a case-control study in a Chinese Han population. A total of 302 GC patients and 300 control individuals were recruited. We identified three single nucleotide polymorphisms (SNPs) in *TERT* that were associated with GC. Odds ratios (ORs) and 95% confidence intervals (CIs) were calculated in logistic regression models after adjusting for age and gender to assess the association. The minor alleles of three SNPs were associated with increased GC risk inallelic model analysis. For two of the SNPs, rs10069690 and rs2853676,, the dominant and additive model frequencies were higher in GC cases compared to controls. Further haplotype analysis revealed a protective effect of haplotype “CG” of the *TERT* gene, while the haplotype “TA” increased GC risk.Our resultsprovide new evidence for the association between *TERT* and GC susceptibility in the Chinese Han population.

## INTRODUCTION

Telomerase, a ribonucleoprotein complex that maintains telomere length at the ends of chromosomes, regulates cellular immortality and tumorigenesis. Normal cells maintain senescence and protect the ends of chromosomes from recombination and end-to-end fusion by shortening telomeres after every cell devision. The *TERT* gene, which encodes the catalytic subunit of telomerase, is expressed in most aggressive cancer cells but is silenced in non-immortalized cells. Re-activation of *TERT* is observed in multiple cancers, indicating that *TERT* is likely driving increased telomerase activity for these malignant cells. This increased activity enables them to overcome replicative senescence and escape apoptosis, and leads to cell immortality [1, 2].

Gastric cancer is a common cancer that is influenced by both genetic and environmental factors. Recent genome-wide association studies (GWAS) have shown that single nucleotide polymorphisms (SNPs) in *TERT* are associated with the risk of multiple cancers. However, few studies in Han Chinese populations have examined the association between *TERT* and the risk for GC. In the current study, we evaluated 3 SNPs in *TERT* associated with GC risk and found a significant association between these SNPS and GC in a Han Chinese population.

## RESULTS

The Chinese Han patient cohort was from Shaanxi province or nearby regions, and comprised of 302 cases and 300 controls. The characteristics of the study population are summarized in Table 1. The ages of controls and cases were 58.01 ± 11.267 and 60.42 ± 5.143 years, respectively, and there were no differences in age distributions between groups. Subjects were genotyped for SNPs in *TERT* to determine whether there was a genetic association with GC. All genotype frequencies in controls conformed to Hardy-Weinberg equilibrium (all *p* > 0.05). Associations between *TERT* genotypes and the risk of gastric cancer are listed in Table 2. We identified two significant SNPs associated with the risk of GC (rs10069690, *p* = 0.021 and rs2853676, *p* = 0.006). The differences in frequency distributions of alleles between cases and controls were compared by Chi-squared test and three SNPs in the *TERT* gene were associated with GC risk at a 5 % level (rs10069690, *p* = 0.004, [OR] = 1.56, 95% [CI] = 1.15−2.11; rs2242652, *p* = 0.017, [OR] = 1.43, 95%[CI] = 1.06−1.91 and rs2853676, *p* = 0.001, [OR] = 1.63, 95% [CI] = 1.21−2.19).

**Table 1:** Age and gender

	case	%	control	%
Total	300		503	
Sex				
Female	69	22.80%	120	40.00%
Male	233	77.20%	180	60.00%
Mean ± SD				
Age	58.01 ± 11.267		60.42 ± 5.143	

**Table 2:** Allele and genotype distributions of SNPs in TERT and their associations with risk of gastric cancer

SNP	Sample	Genotype distribution n			*p*^#^	Allele distribution		OR (95% CI)	*p*^#^	HWE *p*-value
rs10069690		TT	TC	CC		T	C			
	case	18	89	195	0.021*	125	479	1.56 (1.15–2.11)	0.004*	
	control	8	69	219		85	507			0.347
rs2242652		AA	AG	GG		A	G			
	case	17	95	190	0.066	129	475	1.43 (1.06–1.91)	0.017*	
	control	9	78	213		96	504			0.523
rs2853677		GG	GA	AA		G	A			
	case	97	217	173	0.171	231	373	1.25 (0.99–1.58)	0.066	
	control	106	252	144		199	401			0.696
rs2853676		TT	TC	CC		T	C			
	case	16	100	186	0.006*	132	472	1.63 (1.21–2.19)	0.001*	
	control	7	74	219		88	512			0.817

SNP single nucleotide polymorphism, OR odds ratio, 95% CI 95% confidence interval, HWE Hardy–Weinberg equilibrium.

*P*^#^ value from were calculated from two-sided Chi-squared test.

p* ≤ 0.05 indicates statistical significance.

Logistic regression analyses revealed that SNPs rs10069690 and rs2853676 were associated with GC risk by both a dominant model (rs10069690, [OR] = 1.67; 95% [CI] = 1.16−2.4; *p* = 0.006 and rs2853676, [OR] = 1.72; 95% [CI] = 1.2−2.45; *p* = 0.003) and an additive model (rs10069690, [OR] = 1.61; 95% [CI] = 1.19−2.18; *p* = 0.002 and rs2853676, [OR] = 1.63; 95% [CI] = 1.2−2.21; *p* = 0.002; Table 3). All analyses were adjusted by age and gender, and associations with risk of GC remained significant.

**Table 3:** Frequency distributions of prominent SNPs and their associations with the risk of gastric cancer

SNP	Minor allele	MAF		Dominant model		Recessive model		Additive model	
		Case	Control	OR (95% CI)	*p^#^*	OR (95% CI)	*p*^#^	OR (95% CI)	*p*^#^
rs10069690	T	0.207	0.144	1.67 (1.16–2.4)	0.006*	2.62 (1.08–6.34)	0.033*	1.61 (1.19–2.18)	0.002*
rs2242652	A	0.214	0.160	1.49 (1.05–2.12)	0.026*	2.32 (0.98–5.49)	0.054	1.47 (1.09–1.97)	0.011
rs2853677	G	0.382	0.332	1.34 (0.96–1.88)	0.089	1.27 (0.76–2.12)	0.356	1.24 (0.97–1.59)	0.088
rs2853676	T	0.219	0.147	1.72 (1.2–2.45)	0.003*	2.26 (0.89–5.73)	0.085	1.63 (1.2–2.21)	0.002*

*p*^#^ values were calculated by unconditional logistic regression.

p* ≤ 0.05 indicates statistical significance.

**Figure 1:**
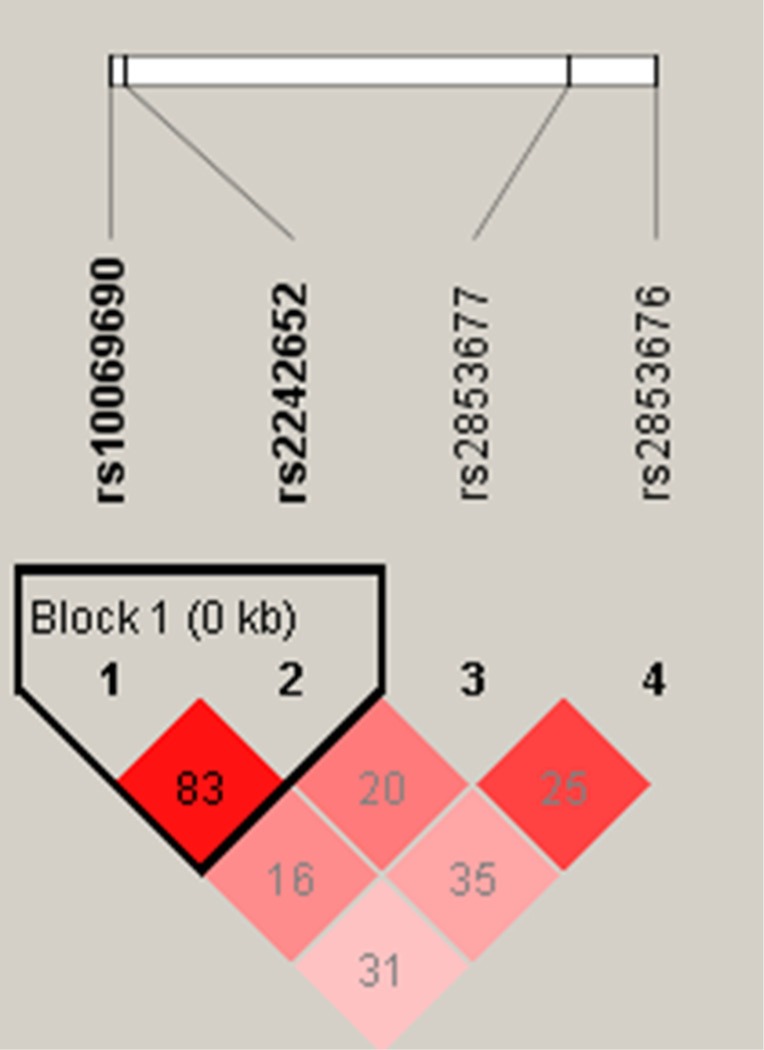
Linkage disequilibrium plots from Chr5p15.33 Red squares indicate statistically significant associations between a pair of SNPs, as measured by D'; darker shades of red indicate a higher D'.

Haplotype analysis was performed for associations between GC and multiple SNPs. Haplotype “TA” was associated with an increased risk of GC ([OR] = 1.346; 95% [CI] = 1.013–1.786; *p* = 0.04), while a protective haplotype “CG” was also detected ([OR] = 0.65; 95% [CI] = 0.4833–0.874; *p* = 0.004) after adjustments for age and gender. The results for the association between the TERT haplotype and the risk of GC are listed in Table 4. In Figure 1, the red squares of the TERT linkage disequilibrium (LD) block exhibited statistically significant linkage between rs10069690 and rs2242652.

**Table 4:** The haplotypes of two SNPs (rs10069690 and rs2242652) and risk of Gastric cancer (adjusted by age and gender)

Haplotype	freq(case)	freq(control)	OR	[95%CI]	*P*^#^
TA	0.197	0.139	1.583	[1.164–2.154]	0.003
CA	0.017	0.017	0.813	[0.329–2.01]	0.654
CG	0.777	0.84	0.65	[0.483–0.874]	0.004

*P*^#^ for logistic regression adjusted by age and gender, *P* < 0.05 indicates statistical significance.

OR: Odds ratio. CI: Confidence interval.

## DISCUSSION

Chromosome 5p15.33, which contains at least two plausible candidate genes, *TERT* and *CLTPM1L*, is a unique cancer susceptibility locus associated with about 10 distinct cancers [3, 4]. telomerase consists of a protein with reverse transcriptase activity encoded by *TERT* and an RNA component that serves as a template for the telomere repeat. *TERT* (also known as *TP2, TRT, CMM9*, *and EST2*) is expressed in several cell types, anditsexpression of *TERT* regulates cellular senescence, the result of the progressive shortening of telomeres with each cell division until they reach a critically short length (http://www.ncbi.nlm.nih.gov/gene/7015). GC patients havehad shorter telomere lengthslength than healthy people, indicating that short telomere length may be associated with increased GC risk.

*TERT*TERT is crucial in activating most immortal cells and cancer cells [5]. Yoo et al. suggested that telomerase activity is detected in 73% of tumors, including gastric carcinomas and non-neoplastic gastric mucosa, while adjacent normal tissues show no enzyme activity [6]. Du et al. examined the role of genetic variants at 5p15 in the progression of multiple cancers, and in GC they found that TERT controls telomere length to cause genomic instability [22]. More recently, several studies have reported that TERT regulates cell proliferation and metastasis, and that it also has a strong effect on alternative splicing and genetic control of telomere length [24, 25]. Telomerase also effects tumor proliferation [26], and Lu et al. demonstrated that re-activation of telomerase activity promoted invasiveness and metastasis [27]. Previous studies have focused on the risk of many cancer types like glioma [8], thyroid [9], melanoma [10], breast [11], ovarian [12], endometrial [13], liver [14] and pancreatic cancer [15], Therefore, we hypothesized that polymorphisms in *TERT* may affect the control of telomere length, resulting in invasion and metastasis of gastric tumors.

We focused on the effects of genetic variants in the Chinese Han population. We tested associations of SNPs in *TERT* with GC risk based on an existing GWAS data [16]. In this case–control study we found that rs10069690, rs2853676, and rs2242652 in the *TERT* gene were associated with an increased risk of GC. TheT allele of rs10069690 in *TERT* was associated with a decreased risk for prostate cancer, and it has also been associated with increasedregulated risks of breast and ovarian cancer [12]. As for SNP rs2853676, the risk was increased in GC patients, confirming previous findings that rs2853676 is associated with increased risk of multiple cancer types [17], including glioma, adenocarcinoma, squamous cell carcinoma, and ovarian cancer [18, 19]. The SNP rs2242652 was previously associated with increased breast cancer risk [12], and both rs2853676 and rs2242652 have been associated with the risk of melanoma [21], but we did not find any significant association of rs2242652 with GC in this study. Haplotype analysis suggested that the combination of certain SNPs could increase or decrease the risk of GC. A protective effect was also observed for the haplotype “CG” of the *TERT* gene that was associated with a 35% reduction in the risk of GC in our study.

Importantly *TERT* may be upregulated in cancer-free patients in precursor lesions of GC. There is also evidence that full-length *TERT* mRNA and protein are expressed in normal gastric samples [30]. High *TERT* expression in intestinal metaplasia and gastric ulcers have been found, suggesting that over-expression of *TERT* may act as an early event in gastric carcinogenesis and that detection of *TERT* could be useful as an early stage marker for the diagnosis of GC [28, 29]. Although the use of *TERT* and telomerase as GC markers is still controversial, that *TERT* will become a useful marker for the early diagnosis of GC. Further investigationsto determine the expression of *TERT* in GC and normal gastric tissues would be helpful.

Despite the statistical power of the current study, there are several limitations. First, study participants were enrolled from the northwest Chinese Han population but the majority of them lived in Shanxi province and its adjacent areas. Thus, future prospective studies are required to confirm these findings and assess their applicability to other ethnic groups. Second, the sample size was relatively small; thus, sequencing of more samples in additional Chinese Han populations and functional assessment of genetic variants are necessary to further validate our findings.

## MATERIALS AND METHODS

### Subjects

The use of human samples in this study was approved by the local Ethics Committees and all participants gave consent for their participation. The cases were GC patients recruited from the Second Department of General Surgery of Shaanxi Province Hospital. All GC patients were diagnosed by expert physicians from the Department of General Surgery based on standard diagnostic criteria. Control subjects were recruited from the health checkup center and all of them visited for an annual health examination. Controls were unrelated, age- and ethnicity-matched healthy individuals who were free of GC at the time of enrollment. At last, 302 GC patients and 300 GC-free controls were recruited among the Chinese Han population.

### Demographics and clinical data

Demographic and detailed personal information were collected by a nurse, including age, sex, ethnicity, residential region, and education status. For GC patients, detailed clinical information was collected through a medical chart review or consultation with treating physicians. At least 5 ml of venous blood was collected from each subject.

### SNP selection and genotyping

All 4 SNPs in the *TERT* gene with minor allele frequencies > 5% in the HapMap (http://www.hapmap.org) Han Chinese population, were associated with GC (rs10069690, rs2242652 and rs2853676). A GoldMag-Mini Purification Kit (GoldMag Co. Ltd. Xian city, China) was performed to extract genomic DNA from whole blood. DNAs were stored at −80°C until analysis. DNA concentrations were measured using a NanoDrop 2000 (Thermo Scientific, Waltham, Massachusetts, USA). Sequenom MassARRAY Assay Design 3.0 software was used to design multiplexed SNP MassEXTEND assay, and SNP genotyping was performed utilizing the Sequenom MassARRAY RS1000 as recommended by the manufacturer. Sequenom Typer 4.0 software was used to perform data management and analyses.

### Statistical analysis

We performed statistical analyses by using Microsoft Excel and SPSS 17.0 (SPSS, Chicago, IL, USA). In this study, all two-sided *p* ≤ 0.05 were considered as statistically significant. Each SNP frequency in the control subjects was tested for deviations from Hardy–Weinberg equilibrium (HWE) by Fisher's exact test. Odds ratios (ORs) and 95% confidence intervals (CIs) were determined using unconditional logistic regression analysis adjusting for age and gender. Allele frequencies and genotype frequencies for each SNP were compared for cases and controls using the Chi-squared test/Fisher's exact test to determine the associations between genotypes and GC risk.

Three genetic models (dominant, recessive, and additive) were accessed using PLINK software (http://pngu.mgh.harvard.edu/purcell/plink/) to estimate ORs for SNP main effects. ORs and 95% CIs were calculated by unconditional logistic regression analyses adjusted for age and sex. Haploview software (version 4.2) and SHEsis software (http://analysis.bio-x.cn/myAnalysis.php) were used to construct haplotype and genetic associations at significant polymorphism loci and to estimate the pairwise linkage disequilibrium (LD), haplotype construction, and genetic association at polymorphism loci.

## References

[1] Suzuki K, Kashimura H, Ohkawa J, Itabashi M, Watanabe T, Sawahata T, Nakahara A, Muto H, Tanaka N (2000). Expression of human telomerase catalytic subunit gene in cancerous and precancerous gastric conditions. Journal of gastroenterology and hepatology.

[2] Shay JW, Wright WE (2005). Senescence and immortalization: role of telomeres and telomerase. Carcinogenesis.

[3] Wang Z, Zhu B, Zhang M, Parikh H, Jia J, Chung CC, Sampson JN, Hoskins JW, Hutchinson A, Burdette L, Ibrahim A, Hautman C, Raj PS (2014). Imputation and subset-based association analysis across different cancer types identifies multiple independent risk loci in the TERT-CLPTM1L region on chromosome 5p15. 33. Human molecular genetics.

[4] Mocellin S, Verdi D, Pooley KA, Landi MT, Egan KM, Baird DM, Prescott J, De Vivo I, Nitti D (2012). Telomerase reverse transcriptase locus polymorphisms and cancer risk: a field synopsis and meta-analysis. Journal of the National Cancer Institute.

[5] Kim NW, Piatyszek MA, Prowse KR, Harley CB, West MD, Ho PL, Coviello GM, Wright WE, Weinrich SL, Shay JW (1994). Specific association of human telomerase activity with immortal cells and cancer. Science (New York, NY).

[6] Yoo J, Park SY, Kang SJ, Kim BK, Shim SI, Kang CS (2003). Expression of telomerase activity, human telomerase RNA, and telomerase reverse transcriptase in gastric adenocarcinomas. Modern pathology.

[7] Yasui W, Tahara H, Tahara E, Fujimoto J, Nakayama J, Ishikawa F, Ide T, Tahara E (1998). Expression of telomerase catalytic component, telomerase reverse transcriptase, in human gastric carcinomas. Japanese journal of cancer research.

[8] Rajaraman P, Melin BS, Wang Z, McKean-Cowdin R, Michaud DS, Wang SS, Bondy M, Houlston R, Jenkins RB, Wrensch M, Yeager M, Ahlbom A, Albanes D (2012). Genome-wide association study of glioma and meta-analysis. Human genetics.

[9] Landa I, Ganly I, Chan TA, Mitsutake N, Matsuse M, Ibrahimpasic T, Ghossein RA, Fagin JA (2013). Frequent somatic TERT promoter mutations in thyroid cancer: higher prevalence in advanced forms of the disease. The Journal of clinical endocrinology and metabolism.

[10] Huang FW, Hodis E, Xu MJ, Kryukov GV, Chin L, Garraway LA (2013). Highly recurrent TERT promoter mutations in human melanoma. Science (New York, NY).

[11] Haiman CA, Chen GK, Vachon CM, Canzian F, Dunning A, Millikan RC, Wang X, Ademuyiwa F, Ahmed S, Ambrosone CB, Baglietto L, Balleine R, Bandera EV (2011). A common variant at the TERT-CLPTM1L locus is associated with estrogen receptor-negative breast cancer. Nature genetics.

[12] Bojesen SE, Pooley KA, Johnatty SE, Beesley J, Michailidou K, Tyrer JP, Edwards SL, Pickett HA, Shen HC, Smart CE, Hillman KM, Mai PL, Lawrenson K (2013). Multiple independent variants at the TERT locus are associated with telomere length and risks of breast and ovarian cancer. Nature genetics.

[13] Carvajal-Carmona LG, O'Mara TA, Painter JN, Lose FA, Dennis J, Michailidou K, Tyrer JP, Ahmed S, Ferguson K, Healey CS, Pooley K, Beesley J, Cheng T (2015). Candidate locus analysis of the TERT-CLPTM1L cancer risk region on chromosome 5p15 identifies multiple independent variants associated with endometrial cancer risk. Human genetics.

[14] Nault JC, Mallet M, Pilati C, Calderaro J, Bioulac-Sage P, Laurent C, Laurent A, Cherqui D, Balabaud C, Zucman-Rossi J (2013). High frequency of telomerase reverse-transcriptase promoter somatic mutations in hepatocellular carcinoma and preneoplastic lesions. Nature communications.

[15] Petersen GM, Amundadottir L, Fuchs CS, Kraft P, Stolzenberg-Solomon RZ, Jacobs KB, Arslan AA, Bueno-de-Mesquita HB, Gallinger S, Gross M, Helzlsouer K, Holly EA, Jacobs EJ (2010). A genome-wide association study identifies pancreatic cancer susceptibility loci on chromosomes 13q22.1, 1q32.1 and 5p15.33. Nature genetics.

[16] Du J, Xu Y, Dai J, Ren C, Zhu C, Dai N, Ma H, Shi Y, Hu Z, Lin D, Shen H, Jin G (2013). Genetic variants at 5p15 are associated with risk and early onset of gastric cancer in Chinese populations. Carcinogenesis.

[17] Wu D, Yu H, Sun J, Qi J, Liu Q, Li R, Zheng SL, Xu J, Kang J (2015). Association of genetic polymorphisms in the telomerase reverse transcriptase gene with prostate cancer aggressiveness. Molecular medicine reports.

[18] Park SL, Fesinmeyer MD, Timofeeva M, Caberto CP, Kocarnik JM, Han Y, Love SA, Young A, Dumitrescu L, Lin Y, Goodloe R, Wilkens LR, Hindorff L (2014). Pleiotropic associations of risk variants identified for other cancers with lung cancer risk: the PAGE and TRICL consortia. Journal of the National Cancer Institute.

[19] Cao JL, Yuan P, Abuduwufuer A, Lv W, Yang YH, Hu J (2015). Association between the TERT Genetic Polymorphism rs2853676 and Cancer Risk: Meta-Analysis of 76,108 Cases and 134,215 Controls. PloS one.

[20] Li G, Jin TB, Wei XB, He SM, Liang HJ, Yang HX, Cui Y, Chen C, Cai LB, Gao GD (2012). Selected polymorphisms of GSTP1 and TERT were associated with glioma risk in Han Chinese. Cancer epidemiology.

[21] Nan H, Qureshi AA, Prescott J, De Vivo I, Han J (2011). Genetic variants in telomere-maintaining genes and skin cancer risk. Human genetics.

[22] Duarte MC, Babeto E, Leite KR, Miyazaki K, Borim AA, Rahal P, Silva AE (2011). Expression of TERT in precancerous gastric lesions compared to gastric cancer. Brazilian journal of medical and biological research = Revista brasileira de pesquisas medicas e biologicas / Sociedade Brasileira de Biofisica [et al].

[23] Low KC, Tergaonkar V (2013). Telomerase: central regulator of all of the hallmarks of cancer. Trends in biochemical sciences.

[24] Hou L, Zhang X, Gawron AJ, Liu J (2012). Surrogate tissue telomere length and cancer risk: shorter or longer?. Cancer letters.

[25] Melin BS, Nordfjall K, Andersson U, Roos G (2012). hTERT cancer risk genotypes are associated with telomere length. Genetic epidemiology.

[26] Jin X, Beck S, Sohn YW, Kim JK, Kim SH, Yin J, Pian X, Kim SC, Choi YJ, Kim H (2010). Human telomerase catalytic subunit (hTERT) suppresses p53-mediated anti-apoptotic response via induction of basic fibroblast growth factor. Experimental & molecular medicine.

[27] Lu MH, Deng JQ, Cao YL, Fang DC, Zhang Y, Yang SM (2012). Prognostic role of telomerase activity in gastric adenocarcinoma: A meta-analysis. Experimental and therapeutic medicine.

[28] Philippi C, Loretz B, Schaefer UF, Lehr CM (2010). Telomerase as an emerging target to fight cancer--opportunities and challenges for nanomedicine. Journal of controlled release.

[29] Gulmann C, Lantuejoul S, Grace A, Leader M, Patchett S, Kay E (2005). Telomerase activity in proximal and distal gastric neoplastic and preneoplastic lesions using immunohistochemical detection of hTERT. Digestive and liver disease.

[30] Li W, Li L, Liu Z, Liu C, Liu Z, Straat K, Bjorkholm M, Jia J, Xu D (2008). Expression of the full-length telomerase reverse transcriptase (hTERT) transcript in both malignant and normal gastric tissues. Cancer letters.

